# USP44 Stabilizes DDB2 to Facilitate Nucleotide Excision Repair and Prevent Tumors

**DOI:** 10.3389/fcell.2021.663411

**Published:** 2021-04-16

**Authors:** Ying Zhang, Imke K. Mandemaker, Syota Matsumoto, Oded Foreman, Christopher P. Holland, Whitney R. Lloyd, Kaoru Sugasawa, Wim Vermeulen, Jurgen A. Marteijn, Paul J. Galardy

**Affiliations:** ^1^Department of Pediatric and Adolescent Medicine, Mayo Clinic, Rochester, MN, United States; ^2^Department of Molecular Genetics, Oncode Institute, Erasmus MC, Rotterdam, Netherlands; ^3^Biosignal Research Center, Kobe University, Hyogo, Japan; ^4^Department of Pathology, Genentech, South San Francisco, CA, United States; ^5^Division of Pediatric Hematology-Oncology, Mayo Clinic, Rochester, MN, United States; ^6^Department of Biochemistry and Molecular Biology, Mayo Clinic, Rochester, MN, United States

**Keywords:** ubiquitin, animal model, DNA damage, deubiquitinating enzyme, tumor supperssor gene

## Abstract

Nucleotide excision repair (NER) is a pathway involved in the repair of a variety of potentially mutagenic lesions that distort the DNA double helix. The ubiquitin E3-ligase complex UV-DDB is required for the recognition and repair of UV-induced cyclobutane pyrimidine dimers (CPDs) lesions through NER. DDB2 directly binds CPDs and subsequently undergoes ubiquitination and proteasomal degradation. DDB2 must remain on damaged chromatin, however, for sufficient time to recruit and hand-off lesions to XPC, a factor essential in the assembly of downstream repair components. Here we show that the tumor suppressor USP44 directly deubiquitinates DDB2 to prevent its premature degradation and is selectively required for CPD repair. Cells lacking USP44 have impaired DDB2 accumulation on DNA lesions with subsequent defects in XPC retention. The physiological importance of this mechanism is evident in that mice lacking *Usp44* are prone to tumors induced by NER lesions introduced by DMBA or UV light. These data reveal the requirement for highly regulated ubiquitin addition and removal in the recognition and repair of helix-distorting DNA damage and identify another mechanism by which USP44 protects genomic integrity and prevents tumors.

## Introduction

Maintenance of the accuracy of encoded genetic information is essential to guard the integrity of genomic identity across cell division and organism reproduction. A wide array of environmental insults leads to physical or chemical DNA damage that must be efficiently recognized and repaired to safeguard against cell dysfunction and disease. The nucleotide excision repair (NER) pathway of DNA repair is responsible for correcting helix-distorting DNA lesions that result from a range of chemical insults (e.g., cisplatin) or exposure to ultraviolet (UV) light (Kamileri et al., 2012; Scharer, 2013; Marteijn et al., 2014; Zhu and Wani, 2017). The recognition of damage in NER varies depending on the location of the lesion, lesions within an actively transcribed region are recognized by the transcription-coupled NER (TC-NER) pathway of NER, while global-genome NER (GG-NER) is capable of recognizing DNA damage throughout the entire genome. The physiological importance of these mechanisms is underscored by the diseases Cockayne syndrome (CS) and xeroderma pigmentosum (XP) that exhibit severe UV hypersensitivity due to defects in the TC-NER and GG-NER pathways, respectively (DiGiovanna and Kraemer, 2012; Natale and Raquer, 2017). While damage in the TC-NER pathway is detected indirectly by the stalling of RNA polymerase II (Fousteri et al., 2006), GG-NER involves the direct detection of damage through the protein products of the genes XP group C (XPC) and XP group E (XPE; DDB2). Cyclobutane pyrimidine dimers (CPDs) and 6-4 photoproducts (6-4PPs) are the predominant lesions introduced by UV light, with CPDs being more abundant and repaired more slowly compared with 6-4PPs. CPDs have also been shown to be the predominant mutagen responsible for the development of UV induced skin cancer in mice (Jans et al., 2005). These lesions are also distinguished by the mechanisms of their detection. While XPC can detect and promote the repair of both CPDs and 6-4PPs *in vitro*, it has a very low affinity for CPDs *in vivo* necessitating a separate pathway for their recognition (Sugasawa et al., 2001; Wakasugi et al., 2002; Scrima et al., 2008). DDB2, as part of the UV-DDB ubiquitin ligase complex that also includes DDB1 and CUL4A, has a high affinity for CPDs *in vivo* and cells with mutated DDB2 have a selective deficit in their repair (Sugasawa et al., 2001). UV-DDB, however, cannot recruit the downstream TFIIH complex that is required to coordinate strand excision. To accomplish this then, DDB2 forms a direct complex with XPC after binding to CPDs (Fitch et al., 2003; Sugasawa et al., 2005; Fei et al., 2011; Matsumoto et al., 2015). Both DDB2 and XPC are substrates of the Ub ligase activity of UV-DDB with differing results. DDB2 ubiquitination leads to reduced affinity for DNA and its proteasomal degradation while ubiquitination of XPC increases its affinity for DNA but does not lead to its destruction (Sugasawa et al., 2005; Matsumoto et al., 2015). The result then of the ubiquitination event is the “pass-off” of CPDs from DDB2 to XPC, which then recruits the TFIIH complex. This model, however, leaves open the question of how DDB2 ubiquitination and degradation is prevented to allow time for XPC recruitment.

We previously demonstrated that the deubiquitinase USP44 is a potent tumor suppressor in mice and that reduced *USP44* levels are also seen in human cancers (Zhang Y. et al., 2012). First characterized as a component of the mitotic spindle assembly checkpoint (Stegmeier et al., 2007), we found that USP44 ensures the timely separation of centrosomes in G2 through a direct complex with the centriole protein centrin. While initially discovered as a component of the centriole structure (Salisbury, 1995), the majority of centrin is not localized to this organelle, instead localizing diffusely through the cytosol and nucleus (Paoletti et al., 1996). Centrin forms a direct complex with XPC, which together with the protein RAD23, forms a trimeric complex that is required for NER activity (Araki et al., 2001; Popescu et al., 2003). USP44 and XPC share a centrin binding motif that is anchored by an invariant tryptophan in both proteins (Zhang Y. et al., 2012). The binding of USP44 with centrin, together with the predominant nuclear localization of USP44 (Zhang Y. et al., 2011; Zhang Y. et al., 2012), led us to question the potential role of USP44 in NER.

Here we show that USP44 is an essential component of NER that is required for the efficient repair of CPDs, but not 6-4 photoproducts. Consistent with the selective CPD repair defect in *Usp44* null cells, we find that USP44 deubiquitinates the recognition protein DDB2 to facilitate its accumulation on DNA lesions and facilitate the subsequent recruitment of XPC. Reinforcing the physiological significance of this mechanism, we find that *Usp44* null mice are hypersensitive to tumors induced by two NER-dependent carcinogens—DMBA and UVB. These findings have important implications for understanding the molecular mechanisms of damage recognition in NER and for the understanding of the tumor suppressive functions of USP44.

## Materials and Methods

### Reagents

CPD and 6-4PP quantitation was performed using commercial ELISA kits (CosmoBio, Carlsbad, CA, United States) according to the manufacturer’s instructions. Antibodies used in this study include anti-DDB2 (#5416), FLAG (DYKDDDDK, #2368), histone H2B (#5546), α-actin (#4970) (Cell Signaling Inc. Danvers, MA, United States), HA (3F10, Millipore-Sigma, St. Louis, MO, United States), V5 (Bethyl Laboratories, Montgomery, TX, United States), CPD (clone TDM-2; CosmoBio) XPC (graciously supplied by Jeff Salisbury) (Acu et al., 2010).

### Mice

All procedures involving mice were reviewed and approved by the Mayo Clinic institutional animal care and use committee (IACUC). Mice carrying hypomorphic or null alleles of *Usp44* were generated and genotyped as previously described (Zhang Y. et al., 2012). The DMBA carcinogen bioassay was performed as described (Serrano et al., 1996). The ultraviolet light tumor assay was performed was adapted from published procedures (Itoh et al., 2004). Briefly, the dorsal skin was shaved twice weekly and the mice were exposed to UVB (312 nm) 2,500 J/m^2^/day on 5 days per week for 20 weeks using a Spectroline E-series lamp equipped with two 8-watt bulbs. Humane endpoints used in addition to standard institutional guidelines include the development of any tumor and moderate-severe dermatitis.

### Cell Lines, Fractionation, Immunoprecipitation

Murine embryonic fibroblasts (MEFs) carrying combinations of hypomorphic or null alleles of *Usp44* were generated and cultured as previously described (Zhang Y. et al., 2012). The human cell line VH10 expressing DDB2-GFP was generated and cultured as previously described (Pines et al., 2012; Ribeiro-Silva et al., 2020). The XPC deficient cell line XP4PA expressing XPC-GFP was generated and cultured as previously described. RNA interference was performed using smart pool reagents from Dharmacon according to manufacturer’s instructions (Hoogstraten et al., 2008; Ribeiro-Silva et al., 2020). Cell fractionation was performed as described (Robu et al., 2013). Immunoprecipitation was performed on extracts generated with 0.1% NP-40, 10% glycerol in PBS as described (Kasper et al., 1999; Zhang Y. et al., 2012).

### Ultraviolet Light Treatment

For quantitative DNA damage repair experiments, cells were washed with PBS and were exposed to 10 J/m^2^ UVC (254 nm) following PBS aspiration using a Mineralight XX-15S lamp (UVP) with two 15-watt bulbs. The exposure time was calibrated using a radiometer (UVX, UVP) prior to each experiment. For subnuclear foci analysis, cells were washed with PBS and a 5 micron micropore filter (Millipore) was overlaid on the cells prior to exposure to 50 J/m^2^ UVC. Cells were fixed with 3% paraformaldehyde at the indicated times after exposure and stained with the antibodies indicated in each figure. The accumulation of DDB2-GFP and the fluorescence recovery after photobleaching (FRAP) of XPC-GFP were performed as described (van Cuijk et al., 2015). The immobilized fraction of XPC-GFP was calculated by comparing the average relative fluorescence intensity measured after recovery was complete (35–45 s after bleaching) expressed as a percentage of the intensity measured prior to bleaching. The relative recovery observed in un-irradiated cells was considered as 100% mobile. The mobility was then similarly calculated in other conditions and normalized to the control cells.

### *In vitro* Deubiquitination Assay

The DDB2-based ubiquitin E3 ligase (CRL-DDB2) was purified as described (Nishi et al., 2005; Sugasawa et al., 2005; Matsumoto et al., 2015). HA-tagged wild-type and catalytic mutant (C281A) USP44 was purified from two 10 cm dishes of MEFs transduced with the respective constructs using the HA tagged protein purification kit (MBL International Corporation, Woburn, MA, United States) according to the manufacturer’s recommendations (Zhang Y. et al., 2012). UbE1 and UBCH5a were obtained from Boston Biochem (Cambridge, MA, United States). CRL-DDB2 (40 ng) was ubiquitinated for 60 min at room temperature (21°C) by incubating UbE1 (200 ng), UBCH5a (800 ng), ubiquitin (10 μg) in a final volume of 30 μl of buffer containing 50 mM Tris-HCl pH 7.6, 10 mM MgCl2, 0.2 mM CaCl2, 4 mM ATP, 1 mM DTT, BSA (3 μg). When this reaction was completed, varying amounts of purified USP44 (2.5, or 5 μl from the 40 μl final volume of protein purified as above) and incubated for an additional 60 min at room temperature (21°C). This reaction was terminated by the addition of SDS sample buffer and boiling.

### Rigor, Reproducibility, Statistics, and Graphing

Unless specified, all experiments were performed at least three times with similar results. All experiments with MEFs were performed with at least three independently derived lines. All immunoblots represent similar findings from at least three independent experiments. Statistical calculations were performed using functions embedded in GraphPad Prism as indicated in each figure legend. Unless specified, all graphs represent the means ± SEM for at least 3 independent experiments. Outlier values were identified and excluded using the Grubbs test at 0.05 alpha. Unless specified, *p*-values were considered significant if <0.05. All graphs were prepared using GraphPad Prism.

## Results

### USP44 Is Required for the Repair of CPDs

**FIGURE 1:**
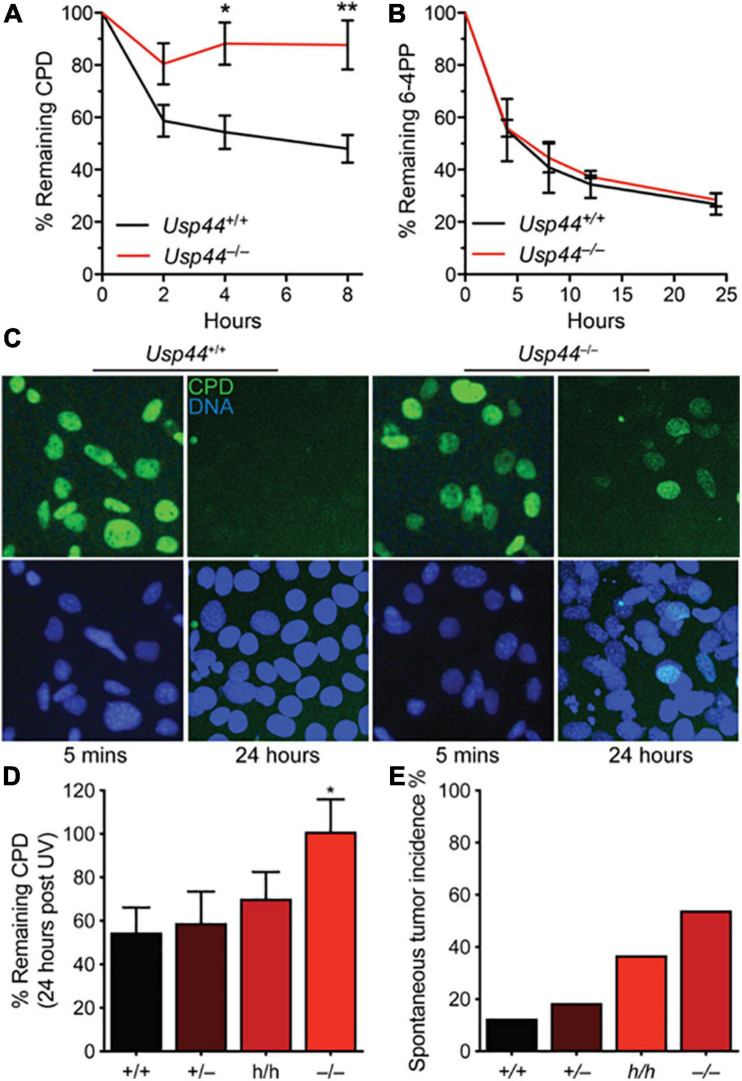
USP44 is selectively required for the repair of cyclobutene pyrimidine dimers. **(A,B)** MEFs of the indicated genotypes were exposed to UVC (10 J/m^2^) and the presence of cyclobutene pyrimidine dimers (CPDs) or 6-4 photoproducts (6-4PPs) were monitored by ELISA at the times shown. The graph represents the mean ± SEM at each time point for three independent MEF lines. **(C)** MEFs from A were stained as indicated 5 min or 24 h after exposure to UVC as in **(A,B)**. **(D)** CPDs were monitored as in **(A)** in MEFs of the indicated genotypes. “*” indicates *p* < 0.05. E. Mice of the indicated genotypes were aged for 18 months and the incidence of tumors detected at necropsy is shown. Note: data for *Usp44* +/+, +/-, and -/- were previously published and included for comparison with the results from *Usp44* h/h mice.

Because of the direct interaction between USP44 and centrin, and the known function of centrin as a cofactor in NER, we asked whether USP44 loss affects the response to UV induced DNA damage. To address this, we exposed wild-type or *Usp44* null MEFs to ultraviolet light (UVC) and monitored the repair kinetics of the predominant UVC induced lesions 6-4 photoproducts (6-4PPs) and CPDs by ELISA. As has been observed by others, 6-4PPs are rapidly repaired in wild-type cells while CPD repair kinetics are slower (Figures 1A,B). Compared to wild-type cells, *Usp44* null MEFs repaired 6-4PPs with normal kinetics, but had a selective defect in the repair of CPDs. Consistent with this finding; we observed strong continued CPD immunostaining of *Usp44* null MEFs 24-h after UVC exposure—a time when most damage has been repaired in wild-type cells (Figure 1C). There was no significant difference in cell survival following UVC exposure, regardless of genotype (data not shown). We next examined the effect of *Usp44* gene dosage on NER through the use of mice carrying a combination of wild-type (+), hypomorphic (h), or null (–) alleles (Zhang Y. et al., 2012). By combining these alleles we generated a series of mice predicted to have graded expression of USP44 and we examined CPD repair in MEFs derived from these animals. There was a strong inverse relationship between the *Usp44* genotype and the amount of CPDs that remained after a 24-h repair period following UV exposure (Figure 1D) though only the difference between wild-type and null is significant. The degree of CPDs remaining bore a striking similarity with the incidence of spontaneous tumors in these mice, though all spontaneous tumors occurred within internal organs similar to that previously reported for *Usp44* null mice (Figure 1E; Zhang Y. et al., 2012).

### USP44 Deubiquitinates and Prevents the Premature Degradation of DDB2

**FIGURE 2:**
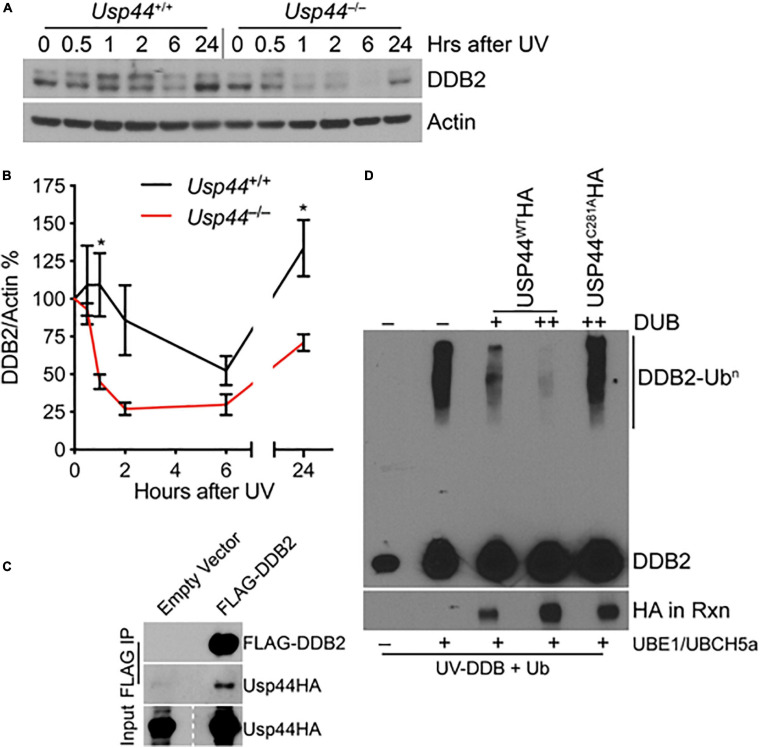
USP44 deubiquitinates DDB2 following UVC exposure. **(A,B)** MEFs of the indicated genotype were exposed to 30 J/m^2^ UVC and samples were collected for immunoblot at the indicated times. The doublet bands were consistently observed in these mouse cells, and were both quantitated to represent DDB2 using imageJ. The graph represents the mean ± SEM for three independent MEF lines. “*” denotes *p* < 0.05. **(C)** MEFs were transduced with the indicated constructs and subjected to immunoprecipitation for the FLAG epitope and probed as indicated. **(D)** Purified CRL-DDB2 was auto-ubiquitinated by the addition of UbE1, UBCH5a, and ubiquitin, and subsequently incubated with either wild type or catalytic mutant (C281A) immunopurified USP44 as indicated. Rxn = reaction.

Because of the selective defect in CPD repair in *Usp44* null cells, we examined the level and degradation kinetics of DDB2 that is required for the *in vivo* repair of CPDs, but not 6-4PPs. As expected, DDB2 levels decline after exposure to UV in wild-type cells with levels returning to—or exceeding baseline levels by 24 h (Figures 2A,B). In comparison, DDB2 underwent a more rapid and profound degradation and failed to recover to wild-type levels in cells lacking USP44. Consistent with a possible enzyme-substrate relationship, we also repeatedly observed USP44 to co-precipitate with DDB2 (Figure 2C), though we were unable to reproducibly demonstrate the reciprocal co-precipitation of DDB2 with USP44. To determine if DDB2 is a direct substrate of USP44, we established an *in vitro* deubiquitination assay. We observed robust ubiquitination of DDB2 when we combined purified CRL-DDB2 with ubiquitin E1 activating enzyme UBE1, the E2 conjugating enzyme UBCH5a, Ub, and ATP (Figure 2D). Following auto-ubiquitination of DDB2, we conducted a second incubation with immunopurified wild-type or mutant USP44. Ubiquitination of DDB2 was reversed by the inclusion of wild-type USP44 but not the catalytically inactive USP44^*C*281A^ (Zhang Y. et al., 2012). These data lead us to conclude that USP44 deubiquitinates DDB2 following UV exposure and prevents its premature degradation.

### USP44 Promotes the Accumulation of DDB2 on Damaged DNA

**FIGURE 3:**
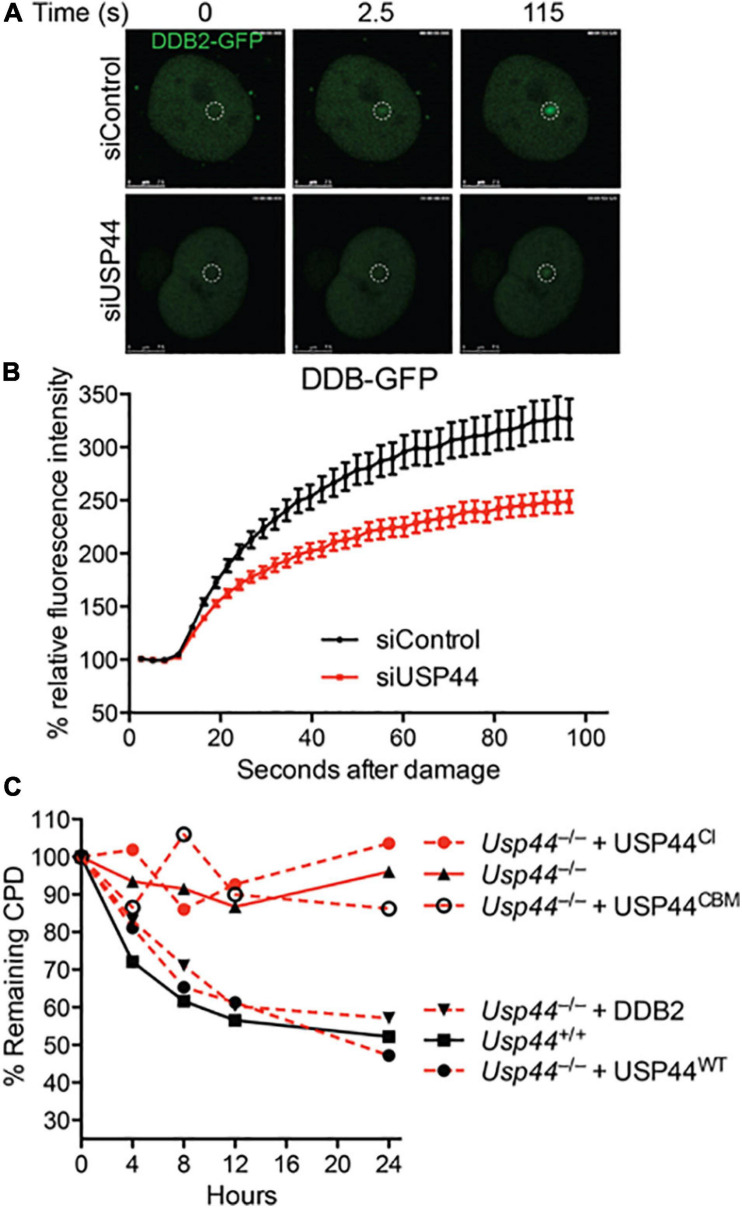
Incomplete CPD repair in *Usp44* null cells associated with inadequate DDB2 recruitment to sites of damage. **(A,B)** VH10 cells expressing DDB2-GFP were transfected with control or USP44 targeting siRNA and then locally irradiated. The DDB-GFP fluorescence was monitored over time using live-cell confocal imaging and quantified to pre-damage intensity set at 100. The graph represents the mean ± SEM for 31–36 cells per condition. **(C)** MEFs of the indicated genotypes were transduced with wild-type, catalytic mutant (USP44^*CI*^; C281A), centrin-binding deficient (USP44^*CBM*^; W162A) USP44, or DDB2 as indicated. The cells were exposed to UVC (10 J/m^2^) and CPD levels were monitored at the indicated times. The graph represents the means of three independent experiments for each condition.

To better understand the impact of USP44 loss on DDB2 localization, we examined the kinetics of DDB2 accumulation on chromatin using live-cell fluorescent microscopy. As the level of DDB2 affects the NER reaction, these experiments were performed in human VH10 fibroblasts expressing DDB2-GFP at near endogenous levels (Pines et al., 2012; Ribeiro-Silva et al., 2020). Subnuclear DNA damage was induced using an UVC laser (266 nm) and the level of DDB2-GFP at the damage was monitored over time. In cells transfected with control siRNA, DDB2-GFP rapidly accumulates at local UV-induced damage sites reaching a plateau approximately 100 s after damage (Figures 3A,B and Supplementary Figure 1). In contrast, cells transfected with USP44 targeting siRNA had a significant defect in the DDB2-GFP accumulation kinetics. This suggests that in the absence of USP44, either DDB2 is more quickly degraded upon binding to DNA damage, or that its ubiquitination prevents efficient chromatin association on damage sites. To further investigate the impact of DDB2 degradation, we examined the ability of various constructs to rescue the CPD repair defect in a complementation assay. As expected, reintroducing wild-type USP44, but not the catalytically inactive USP44^*C*281A^ (Zhang Y. et al., 2012) completely restored CPD repair in *Usp44* null MEFs (Figure 3C). Similarly, and demonstrating a requirement for the USP44-Centrin complex, the centrin binding mutant USP44^*W*162A^ (which is fully catalytically active; Zhang Y. et al., 2012) failed to rescue CPD repair. Overexpressing FLAG-DDB2 completely restored CPD repair in *Usp44* null MEFs, consistent with it being a limiting factor in cells lacking USP44. We conclude that the rapid degradation of DDB2 upon DNA damage is responsible for the defective repair of CPDs in *Usp44* null MEFs.

### XPC Recruitment to DNA Damage Is Impaired in the Absence of USP44

**FIGURE 4:**
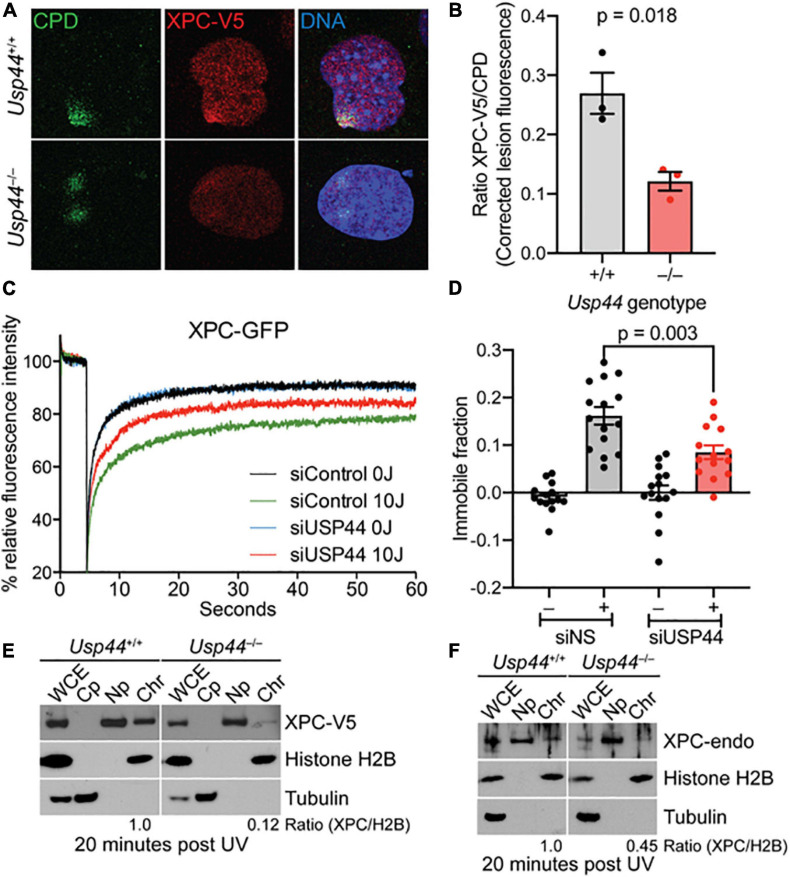
Recruitment of XPC to UV induced DMA damage sites requires USP44. **(A,B)** MEFs of the indicated genotype fixed and stained as indicated 20 min after exposure to UVC (50 J/m^2^) through a micropore filter (5 μM), The intensity of XPC to CPD lesions was quantitated using imageJ and was normalized to the intensity of the corresponding CPD. The graph represents the mean of the means from three independent experiments, *n* = 64–65 individual lesions total per genotype. **(C)** XPC deficient human fibroblasts expressing XPC-GFP were transfected with control or USP44 targeting siRNA. After exposure (or not) to UVC, cells were photobleached and the recovery of fluorescence was monitored by live-cell fluorescence microscopy, plotted relative fluorescence was normalized to prebleach values set at 100. Note the reduced recovery of control cells reflecting XPC immobilization and the enhanced recovery seen in USP44 depleted cells. The graph represents the mean of 15 cells per condition. **(D)** Immobile fractions of XPC-GFP in cells transfected with the indicated siRNA and analyzed by FRAP in **(C)**. **(E,F)** MEFs of the indicated genotypes were exposed to UVC (30 J/m^2^)and cells were harvested and separated into fractions as indicated (WCE, whole cell extract; Cp, cytoplasm; Np, nucleoplasm; Chr, chromatin). The presence of V5-tagged XPC **(D)** or endogenous XPC **(E)** in the chromatin fraction relative to histone H2B (chromatin marker) was determined using image J, The blots are representative of 2–3 independent experiments of each type.

While DDB2 is important for the initial recognition of CPDs, the “hand-off” of these lesions to XPC is ultimately required for their repair. To examine the impact of USP44 loss on XPC recruitment we examined the colocalization of XPC with CPDs by confocal immunofluorescence microscopy. As we observed with DDB2, there was a decrease in the localization of XPC on CPD lesions in *Usp44* null MEFs compared with wild-type (Figures 4A,B). To further investigate this, we again used live-cell fluorescence microscopy. *XPC* deficient human fibroblasts expressing endogenous levels of XPC-GFP were analyzed with fluorescence recovery after photobleaching (FRAP) to examine the immobilization of XPC on chromatin after UVC exposure. In control cells, as expected, exposure to UVC leads to a strong immobilization of XPC indicating increased residence time at sites of damage. Comparing control cells with those depleted of USP44, showed a reduction in the UV-induced XPC immobilization, indicating impaired binding of XPC at damage sites (Figures 4C,D). This is particularly noteworthy as only the immobilization of XPC on CPDs would be expected to be impaired in the absence of USP44. Deficient XPC binding to chromatin was confirmed by subcellular fractionation in wild-type or *Usp44* null MEFs. We observed a decreased amount of XPC-V5 in the chromatin fraction in USP44 null MEFs 20 min after UVC exposure (Figure 4E). Similar results were also obtained by assessing the chromatin binding of endogenous XPC (Figure 4F). Taken together these data indicate that USP44 assists in the recruitment of DDB2 to damaged chromatin and that this defective DDB2 accumulation results in reduced XPC localization to these lesions.

### USP44 Is Required to Protect Mice From Tumors Following NER Pathway Carcinogens

**FIGURE 5:**
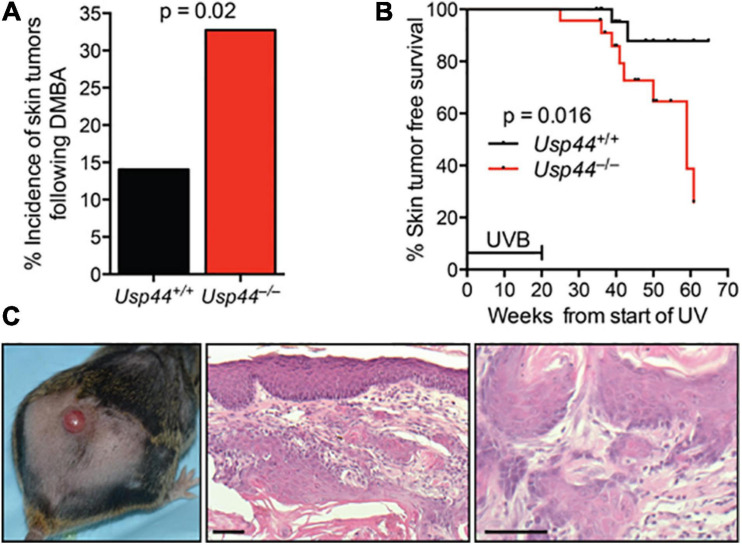
*Usp44* null mice have increased sensitivity to UVB-induced skin tumors. **(A)** Newborn pups (1–3 days of live; n = 55–57 per genotype) were exposed to a single topical dose of DBMA. Mice were monitored for 5 months at which point the incidence of skin tumors was determined. **(B)** Mice of the indicated genotypes (*n* = 25 each) were exposed to UVB (2,500 J/m^2^/day) 5 days per week for 20 weeks. The incidence of skin tumors was monitored. The graph represents the Kaplan-Meier estimated skin tumor free survival for the cohorts. **(C)** Representative images of skin -gross (left) H&E stained (right) of tumors observed in *Usp44* null mice from **(B)**. Scale bar = 50 μm.

To examine the impact of the identified NER defect on tumorigenesis, we exposed pups of *Usp44*^±^ intercrosses to a single dose of the carcinogen DMBA that induces DNA adducts that are repaired by NER (Wijnhoven et al., 2001). Animals were scored for tumors at 5 months of age. We found a significant increase in skin tumors in *Usp44* null mice compared with wild-type (Figure 5A). To further examine the role of USP44 in preventing DNA damage induced tumors, wild-type or *Usp44* null mice were exposed to UVB for 5 days per week for 20 weeks and were monitored for skin tumors. There was a significant increase in the rate of skin tumors in *Usp44* null mice (Figures 5B,C). Of note, there were also a number of internal tumors that developed in *Usp44* null mice during the observation period, including some discovered in moribund animals. As these do not appear to be metastatic tumors from the skin (no skin tumors identified in these mice) it seems unlikely that these are related to the UVB exposure. However, we have not otherwise observed life-threatening tumors in the first year of life in *Usp44* null mice. We conclude that the NER activity of USP44 is important in its ability to suppress tumors resulting from UV exposure.

## Discussion

Targeted ubiquitination plays an important role in many aspects of the repair of DNA damage, including lesion recognition and repair during the NER pathway. Based on our observation that USP44 binds the XPC partner centrin, we hypothesized that it played a role in NER. Our results demonstrate that USP44 is an important component of this pathway and is required specifically for the repair CPDs—a lesion that is dependent on recognition by DDB2. Based on our data, we envision a model in which USP44 counteracts the UV-induced DDB2 ubiquitylation. After UV exposure, DDB2 binds to CPDs on chromatin where it becomes auto-ubiquitinated. This ubiquitination lowers the affinity of DDB2 for DNA and results in its dissociation and degradation. Based on the data presented in this manuscript USP44 could be involved in two processes. One possibility is that USP44 de-ubiquitinates DDB2 once it has been released, allowing it to be recycled back onto persisting CPDs. In cells proficient for USP44, the DDB2 is rapidly de-ubiquitinated, resulting in an increase protein lifetime, allowing efficient re-association with chromatin. DDB2 recruits XPC to CPDs that it cannot efficiently recognize itself. Therefore, in the presence of USP44, the de-ubiquitination of DDB2 allows it to iteratively cycle back onto damaged chromatin until XPC is recruited. In contrast, cells lacking USP44 cannot recycle the released DDB2 back onto the chromatin—leading to reduced DDB2 residency on CPDs, impairing recruitment of XPC and the repair of these lesions.

A second scenario is that USP44 may remove ubiquitin from DDB2 bound to CPDs. This would suppress DDB2 dissociation and degradation and enhance XPC recruitment. The lack of USP44 enrichment at sites of localized DNA damage might suggests it preferentially acts in the soluble phase to stabilize damage-evicted DDB2, rather than acting directly at damaged chromatin. However, as DUB-substrate interactions may be transient, we cannot exclude a role of USP44 at chromatin bound DDB2. Data supporting this model is the finding that USP44 stimulates DDB2 accumulation directly upon DNA damage induction (Figure 3B). The severe reduction of DDB2 accumulation kinetics at damage sites in living cells is most consistent with a direct effect of USP44 on chromatin-bound DDB2 rather than enhanced recycling. In compliance with this latter model, it was recently found that XPC competitively suppresses ubiquitination of DDB2 and this effect is significantly promoted by centrin-2, which may be explained by the recruitment of USP44 via its centrin binding domain (Matsumoto et al., 2015). In line with this, our data demonstrate the requirement for centrin binding by USP44, suggesting that DDB2 is de-ubiquitinated while bound at the UV-induced lesion by XPC-centrin recruited USP44. Importantly, USP44 may be involved in both mechanisms, by de-ubiquitinating chromatin bound DDB2 and by stabilizing the damage-evicted DDB2, both important for efficient XPC damage binding (Fig XX). While mechanistic details remain, our data provide compelling evidence that USP44 stabilizes DDB2 and that this mechanism is crucial for efficient XPC recruitment and has physiological relevant roles in tumor prevention.

As CPDs represents only one DDB2-dependent substrate repaired through NER, and given that we lack a clear understanding of other DDB2-dependent lesions, it remains possible that USP44 may be involved more generally in facilitating DNA damage repair through this pathway. In support of this notion, the increased susceptibility to DMBA induced tumors is not explained by the accelerated destruction of DDB2. *Ddb2* or *Xpc* deficient mice do not have increased tumor susceptibility following DMBA exposure. In contrast, mice lacking the *Ercc6* gene critical for early steps of transcription-coupled NER, as well as components involved in damage verification (*Xpa*) downstream of both the GG- and TC-NER pathways lead to increased DMBA sensitivity (Nakane et al., 1995; van der Horst et al., 1997; de Boer et al., 1998). This may suggest USP44 also participates in the de-ubiquitination in these other NER events. It is also noteworthy in this regard that mouse models lacking XPC and/or DDB2 are prone to tumors in internal organs in addition to being sensitive to UV-induced skin tumors (Itoh et al., 2004; Hollander et al., 2005; Yoon et al., 2005). Recently, DDB2 was found to be a critical component of the base excision repair of 8-oxo-guanine and abasic sites (Jang et al., 2019), potentially accounting for these observations. Additional evidence for NER protecting against internal tumors comes from the large number of genome association studies that have linked polymorphisms in several NER pathway genes with the development or therapeutic response of non-skin tumors (Matakidou et al., 2006; Qiu et al., 2008). This likely reflects the defective repair of other forms of DNA damage (e.g., environmental chemical adducts or oxidative lesions). The additional role of USP44 in the prevention of aneuploidy makes it difficult to say whether the tumors seen in the internal organs of *Usp44* null mice are related to NER defects, particularly since mutation induced tumors may be accelerated through the loss of heterozygosity induced by aneuploidy.

Although it has been known for some time that DDB2 undergoes ubiquitin-dependent degradation after UV exposure, there has been recent attention on understanding how this process may be regulated. Recently, DDB2-dependent poly(ADP-ribosyl)ation (PARylation) at DNA damage lesions was identified (Pines et al., 2012; Robu et al., 2013). One of the targets of this modification was found to be DDB2 itself. As PARylation and ubiquitination occur on lysine residues and both modifications cluster in the N-terminal region of DDB2, a model of PARylation competing for ubiquitination sites was forwarded. In support of this model, PARP inhibition led to increase ubiquitination and accelerated degradation of DDB2. Surprisingly, PARP inhibition had no impact on DDB2 accumulation at damage loci. Whether there are other mechanisms that regulate the timing of DDB2 ubiquitination and degradation is not known.

De-ubiquitinating enzymes play other roles in NER. The best described is USP7 (HAUSP) that is involved both in the TC-NER and GG-NER pathways. USP7 was identified to be the primary enzyme removing ubiquitin from XPC following its ubiquitination by UV-DDB (He et al., 2014), and subsequently was found to be critical for maintaining levels of ERCC6 in the early steps of the TC-NER pathway (Schwertman et al., 2012). Additionally, recent reports identify another DUB, USP24 that plays a role in deubiquitinating DDB2 (Zhang L. et al., 2012). USP24 does not seem to be an essential component of the NER machinery, however, as CPD repair was found to be unperturbed in cells depleted of USP24. This may be due to USP24 not influencing the rate of DDB2 degradation following UV, but rather only at steady state. Interestingly, we find that in the absence of USP44 the levels of DDB2 are preserved at baseline but decline rapidly after UV exposure. This suggests that these DUBs act on DDB2 at different phases of its function in a non-redundant fashion. In another recent report, XPC was found to negatively affect the ubiquitination of DDB2—an effect amplified in the presence of centrin (Matsumoto et al., 2015). The model arising from this data is that the presence of XPC-HR23-Centrin diverts UV-DDB mediated ubiquitination away from DDB2 onto XPC. The proposed model further postulates that the ubiquitin-independent dissociation of DDB2 from chromatin allows its persistence for subsequent rounds of CPD recognition prior to its ultimate degradation. This model is not mutually exclusive with our work as we propose that DDB2 remains ubiquitin-free in part due to the activity of USP44 until XPC arrives, and that its activity may further facilitate the survival of DDB2 for subsequent rounds of damage recognition.

We previously reported the tumor prone phenotype of *Usp44* null mice in the context of its role in the protection against aneuploidy (Zhang Y. et al., 2012). Here we now demonstrate a second distinct tumor suppressive function of USP44. While our current data do not allow us to determine which if either of these roles is most important in its role as tumor suppressor, it raises the intriguing possibility that in fact these functions act in concert to protect against tumors. Aneuploidy is thought to drive tumors, at least in part, through its ability to promote the loss of heterozygosity at the loci of mutated tumor suppressor genes (Baker et al., 2009). The missegregation of chromosomes in mitosis may lead to the loss of the remaining wild-type tumor suppressor locus. In some instances, this may even be followed by the subsequent gain of an additional copy of the chromosome carrying the mutated gene, resulting in so-called copy neutral loss of heterozygosity, a process that also results in uniparental disomy. This model is predicated on the pre-existence of mutated tumor suppressor loci on which the chromosome missegregation may act. It is easy to appreciate how the loss of USP44, resulting in both increased mutagenesis and increased chromosome missegregation, may therefore act as a powerful tumor suppressor. Future work is needed to separate these functions and to determine their relative importance.

## Data Availability Statement

The raw data supporting the conclusions of this article will be made available by the authors, without undue reservation.

## Ethics Statement

The animal study was reviewed and approved by the Mayo Clinic Institutional Animal Use and Care Committee.

## Author Contributions

PG: conceptualization, formal analysis, writing – original draft preparation, project administration, and funding acquisition. YZ, IM, SM, KS, WV, JM, and PG: methodology. YZ, IM, SM, OF, CH, and WL: investigation. SM and KS: resources. YZ, IM, SM, OF, CH, WL, KS, WV, JM, and PG: writing – review and editing. YZ, IM, KS, JM, and PG: visualization. KS, WV, JM, and PG: supervision. All authors have read and agreed to the published version of the manuscript.

## Conflict of Interest

PG owns stock in Abbott laboratories, Abbvie, and Johnson and Johnson. OF is employed by the company Genentech. These companies had no influence on the design or interpretation of the study—nor the writing of the manuscript. The remaining authors declare that the research was conducted in the absence of any commercial or financial relationships that could be construed as a potential conflict of interest.
