# Supplementary figures and images for: USP44 Stabilizes DDB2 to Facilitate Nucleotide Excision Repair and Prevent Tumors
Source: Front Cell Dev Biol. 2021 Apr 16;9:663411. doi: 10.3389/fcell.2021.663411 (PMC8085418; doi:10.3389/fcell.2021.663411)

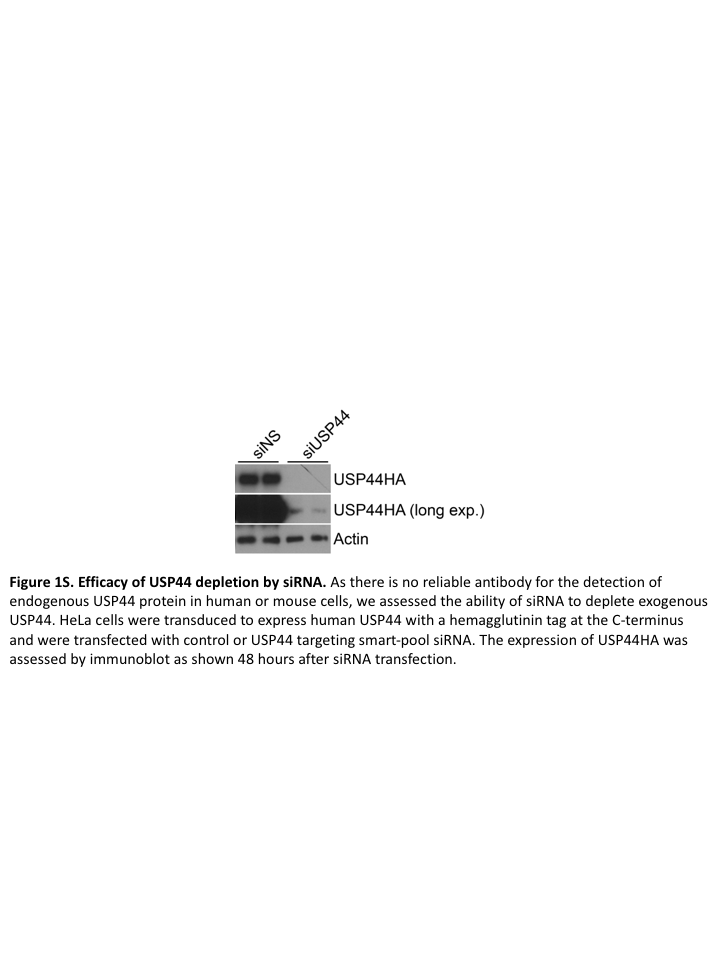

Supplement: Supplementary file 1 [file Image_1.TIFF]
